# An Extensible Evaluation Framework Applied to Clinical Text Deidentification Natural Language Processing Tools: Multisystem and Multicorpus Study

**DOI:** 10.2196/55676

**Published:** 2024-05-28

**Authors:** Paul M Heider, Stéphane M Meystre

**Affiliations:** 1 Biomedical Informatics Center Medical University of South Carolina Charleston, SC United States; 2 Institute of Digital Technologies for Personalised Healthcare (MeDiTech) University of Applied Sciences and Arts of Southern Switzerland Lugano Switzerland

**Keywords:** natural language processing, evaluation methodology, deidentification, privacy protection, de-identification, secondary use, patient privacy

## Abstract

**Background:**

Clinical natural language processing (NLP) researchers need access to directly comparable evaluation results for applications such as text deidentification across a range of corpus types and the means to easily test new systems or corpora within the same framework. Current systems, reported metrics, and the personally identifiable information (PII) categories evaluated are not easily comparable.

**Objective:**

This study presents an open-source and extensible end-to-end framework for comparing clinical NLP system performance across corpora even when the annotation categories do not align.

**Methods:**

As a use case for this framework, we use 6 off-the-shelf text deidentification systems (ie, CliniDeID, deid from PhysioNet, MITRE Identity Scrubber Toolkit [MIST], NeuroNER, National Library of Medicine [NLM] Scrubber, and Philter) across 3 standard clinical text corpora for the task (2 of which are publicly available) and 1 private corpus (all in English), with annotation categories that are not directly analogous. The framework is built on shell scripts that can be extended to include new systems, corpora, and performance metrics. We present this open tool, multiple means for aligning PII categories during evaluation, and our initial timing and performance metric findings. Code for running this framework with all settings needed to run all pairs are available via Codeberg and GitHub.

**Results:**

From this case study, we found large differences in processing speed between systems. The fastest system (ie, MIST) processed an average of 24.57 (SD 26.23) notes per second, while the slowest (ie, CliniDeID) processed an average of 1.00 notes per second. No system uniformly outperformed the others at identifying PII across corpora and categories. Instead, a rich tapestry of performance trade-offs emerged for PII categories. CliniDeID and Philter prioritize recall over precision (with an average recall 6.9 and 11.2 points higher, respectively, for partially matching spans of text matching any PII category), while the other 4 systems consistently have higher precision (with MIST’s precision scoring 20.2 points higher, NLM Scrubber scoring 4.4 points higher, NeuroNER scoring 7.2 points higher, and deid scoring 17.1 points higher). The macroaverage recall across corpora for identifying names, one of the more sensitive PII categories, included deid (48.8%) and MIST (66.9%) at the low end and NeuroNER (84.1%), NLM Scrubber (88.1%), and CliniDeID (95.9%) at the high end. A variety of metrics across categories and corpora are reported with a wider variety (eg, *F*_2_-score) available via the tool.

**Conclusions:**

NLP systems in general and deidentification systems and corpora in our use case tend to be evaluated in stand-alone research articles that only include a limited set of comparators. We hold that a single evaluation pipeline across multiple systems and corpora allows for more nuanced comparisons. Our open pipeline should reduce barriers to evaluation and system advancement.

## Introduction

### Background

An ironclad pillar of clinical data reuse is the proper protection of protected health information (PHI). Deidentification is the process of tagging and removing personally identifiable information (PII) to prevent incidental privacy breaches. Unfortunately, manual deidentification is an expensive and error-prone process [1,2], and automated deidentification remains an unsolved challenge [3,4]. Since the first published automated deidentification system [5], a variety of systems using a range of technologies have been released. In tandem, a series of competitions have been organized around shared corpora annotated with PII to further encourage the development of deidentification systems [6-9]. Researchers publishing about a new system tend to release comparative performance metrics against 1 or 2 publicly available systems using 1 or 2 corpora [3,10-12]. These stand-alone research articles that only include a limited set of comparator systems and corpora cannot always even be directly compared to create a single meta-analysis because of the differences in annotation categories evaluated and reported on or how exactly matches are aligned and scored. Replication of the process to confirm results and extension of the process to evaluate new systems or corpora have been stymied by a combination of lack of documented evaluation code, closed evaluation tools, and incompatible PII categories, among other reasons.

As such, a single evaluation pipeline across multiple systems and corpora allows for more nuanced comparisons between systems. A single pipeline allows researchers (and staff scientists responsible for evaluating systems before deployment) to consistently and reproducibly generate scores for a range of systems across a range of corpora using the exact same methods. Researchers with preferences to evaluate deidentification systems at the character level versus token level versus PII mention can run each evaluation in turn. Similarly, researchers can differentiate or collapse PII categories as desired across all systems and corpora at once to get a clear picture of how each system and corpus, respectively, treats different categories. Not all categories of PII are equally sensitive [3]. Patient names are more revealing than provider names, which are, in turn, more revealing than hospital names.

### Objectives

To that end, we evaluated PII extraction performance at multiple levels of granularity because these systems should not be judged on a single summary performance metric. For instance, this pipeline provides an easy means for surfacing the false negative rate or recall (also called sensitivity) for patient names as distinct from provider names (or other names). We can also easily compare performance when differences between PII categories are important to maintain, as opposed to when all PII categories are treated interchangeably.

As a case study, to help us understand the performance trade-offs of available deidentification systems that are critical to clinical data reuse and natural language processing (NLP) and to foster building larger repositories of directly comparable evaluation results, we developed a reusable and extensible pipeline for evaluating 6 off-the-shelf deidentification systems across 2 freely available corpora, 1 previously available corpus, and 1 private corpus. The systems and corpora all use English, although the pipeline is language agnostic. As none of the systems or corpora use exactly the same annotation schema, we provided mappings to allow approximately equitable performance metrics across all components. Any deidentification system programmatically runnable from the command line can be added to the set. Similarly, new corpora can be added to the evaluation process with minimal constraints on their format or annotation categories. Furthermore, the evaluation tool used in the pipeline allows for analysis at configurable levels of annotation category granularity and with multiple text annotation matching styles [13,14]. We focused on off-the-shelf systems for 3 primary reasons. First, off-the-shelf systems have the lowest barrier to entry. Not all potential users have the skills, capacity, or annotated corpora available to retrain a model. If a deidentification system developer considers their tool to be unusable without retraining, then that limitation should be made explicit, which brings us to the second reason. Undertaking the controlled experiments required to determine when a retrained system has met a reliable and safe performance threshold requires a test harness exactly like the one we propose here. Third, the potential variants for retraining any given system to optimize its performance for a local site constitutes its own large undertaking and falls outside the scope of this research. Furthermore, comparing the retrained variants to determine optimal performance is best organized using a test harness as described in this study.

Thus, we hold that a single evaluation pipeline across multiple systems and corpora allows for the nuanced comparisons required for safe deployment of NLP systems, in general, and deidentification systems, in particular. We document the use and extensibility of such a tool with deidentification as a use case. In summary, we found an order of magnitude difference between the fastest and slowest systems in terms of processing speed. We found that none of the 6 systems consistently outperformed the others across corpora and PII tag categories. A nuanced comparison of the top performers under slightly different conditions would be much more difficult without a cohesive framework like the one we describe.

## Methods

### Ethical Considerations

This study was assessed by the Medical University of South Carolina (MUSC) Institutional Review Board for Human Research (IRB) and officially considered Not Human Research. It was therefore not subject to oversight by the MUSC IRB, since it met the criteria set forth by the Code of Federal Regulations (45CFR46): (1) the data were not collected specifically for the currently proposed research project through an interaction or intervention with living individuals and (2) investigators including collaborators on the proposed research cannot readily ascertain the identity of the individuals to whom the coded private information of specimens pertains.

The other three corpora used in this study (ie, 2006 i2b2 shared task, 2014 i2b2 and University of Texas Health Science Center at Houston (i2b2/UTHealth), and 2016 Centers of Excellence in Genomic Science Neuropsychiatric Genome-Scale and RDOC Individualized Domains [CEGS N-GRID] shared task) were publicly available and already deidentified, and therefore not subject to IRB approval requirements.

### PII Categories

**Figure 1:**
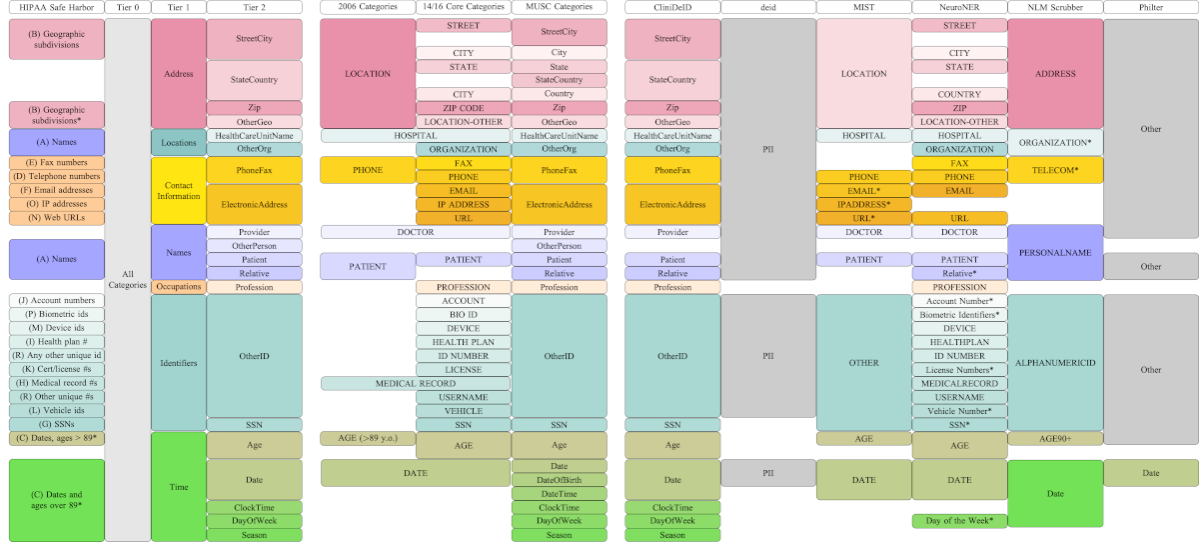
Corpus and system category alignment. Each column represents the categories relevant to HIPAA (Health Insurance Portability and Accountability Act) Safe Harbor Guidelines (column 1), a specific analysis tier (columns 2-4), corpus (columns 5-7), or system (columns 8-13). Each row represents approximate equivalency between categories. An asterisk by a category label (eg, “Relative*”) indicates that the category is attested in the system documentation but not labeled by the system in any corpus. BIO ID: Biometric Identifier; i2b2: Informatics for Integrating Biology and the Bedside; MIST: MITRE Identity Scrubber Toolkit; MUSC: Medical University of South Carolina; NLM: National Library of Medicine; OtherGeo: other geographic subdivision; PII: Personally Identifiable Information; SSN: Social Security Number.

**Figure 2:**
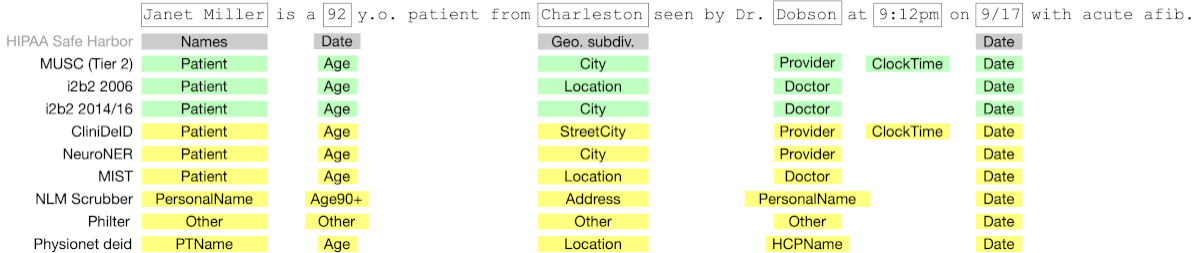
Personally identifiable information terms in sample sentence mapped to categories across all corpora and deidentification systems. Geo. subdiv.: geographic subdivision; HCPName: Health Care Provider Name; HIPAA: Health Insurance Portability and Accountability Act; i2b2: Informatics for Integrating Biology and the Bedside; MIST: MITRE Identity Scrubber Toolkit; MUSC: Medical University of South Carolina; NLM: National Library of Medicine; PTName: patient name.

In the United States, HIPAA (Health Insurance Portability and Accountability Act) is the primary legal mandate guiding and governing data privacy and security provisions within the health care domain [15]. Other regions of the world have enacted similar privacy laws such as the General Data Protection Regulation in the European Union [16], although they are not all specific to the health care industry, as evidenced by the General Data Protection Regulation. The HIPAA privacy rule specifies sensitive classes of PII that should be removed for the data to be considered deidentified. In this study, we focus on the subset of HIPAA’s 18 categories of PII relevant to unstructured clinical notes, as all our data sets were created in the United States under the jurisdiction of HIPAA. We have listed a curated and organized set of these categories in Figure 1 in the 4 left-most columns. The first column indicates the original HIPAA Safe Harbor category names. The following 3 columns are curated classes to help group or simplify the categories. We call these the tier 0 category (which represents the general class of PII), the tier 1 categories (which represent 7 high-level sets of categories grouped by semantic domain and common textual realizations), and tier 2 categories (which represent the finest-grained division of categories approximating the original HIPAA categories). The tier 2 categories include several practical and functional extensions of the strict HIPAA categories that have been treated as PII by deidentification researchers in the clinical domain. For instance, HIPAA considers ages >89 years to be PII but not the not younger ages, while some researchers consider any age to be PII. Additional columns in this figure represent categories annotated in deidentification corpora (columns 5-7) and categories flagged by deidentification systems (columns 8-13). Rows across the figure indicate rough equivalency of categories between tiers, corpora, or systems. Specific details of corpus and system categories are covered in the next two subsections: *Deidentification Shared Tasks* and *Corpora and Deidentification Systems*. Perfect category synchrony between corpora and annotations is impossible. We have included a sample sentence in Figure 2, cross-annotated according to the specifics of each category schema to help highlight the local variation between corpora and deidentification systems.

### Deidentification Shared Tasks and Corpora

Several shared tasks organized around the goal of evaluating deidentification systems have been organized within the clinical domain. The 2006 Informatics for Integrating Biology and the Bedside (i2b2) shared task was the first such task and focused on a small set of PII categories present in unstructured clinical notes written in English [6]. A similar shared task was organized as part of the 2012 NII Test Beds and Community for Information Access Research Medical Natural Language Processing task for fabricated but realistic medical reports written in Japanese [7]. Two more English language tasks were organized with an array of PII categories that were more representative of the full list of HIPAA categories as part of the 2014 i2b2 and UTHealth shared task [8] and 2016 CEGS N-GRID shared task [9].

We used each of the 3 English language deidentification shared task corpora for our study as they have been used as the standard reference corpora in the domain. The 2006 and 2014 corpora are publicly available with an appropriate data use agreement. The 2016 corpus was publicly available but has since been removed from circulation due to privacy concerns. The fourth corpus, also in English, is called the “Medical University of South Carolina (MUSC)” corpus in this study as it was developed at MUSC by the authors. It is not publicly available as its PII has only been annotated and not redacted or resynthesized to prevent the release of PHI.

We refer to the oldest corpus in our study as “2006” as it was used for the 2006 i2b2 shared task [6]. It consists of 889 discharge summaries from Partners HealthCare. This corpus has the most reduced set of annotation categories with 8 distinct categories: location, hospital, phone, doctor, patient, medical record, age (>89 years), and date, as shown in Figure 1. Of note, the tier 1 category that we call “Address” maps to the 2006 category “Location.” “Medical Records” are the only recorded tier 1 “Identifiers.” “Age” is confined by the strict HIPAA notion to those >89 years, in contrast to the other 3 corpora that treat all ages as PII.

Internally, we aligned the annotation categories for the 2014 i2b2 and UTHealth [8] and the 2016 CEGS N-GRID shared tasks [9] on deidentification, which we refer to as “2014” and “2016,” respectively. In contrast with 2006, the tier 1 Address and Identifiers categories for 2014 and 2016 are split into fine-grained categories. Overall, there are 28 distinct categories, as shown in Figure 1. The 2014 corpus consists of 1304 discharge summaries and correspondences between providers from Partners HealthCare. The 2016 corpus consists of 1000 psychiatric notes from Partners HealthCare.

The MUSC corpus consists of 728 notes split across 8 note types: consults, discharge summaries, history and physicals, nursing, pathology and cytology, patient instructions, plan of care, and progress notes. Similar to the 2014 and 2016 corpora, the MUSC corpus includes a wide range of tier 1 “Identifiers” but groups all of them except social security numbers into 1 class: “Other ID.” Due to a change in the annotation guidelines over the course of the corpus annotation, some “Street” annotations and “City” annotations have been merged into a single “StreetCity” annotation. Similarly, some “State” annotations and “Country” annotations have been merged into “StateCountry” annotations.

All 4 corpora have been divided into train and test splits. None of the systems have used the official test splits for training. Therefore, we report all performance metrics with respect to the official test split in the main body of this paper. Multimedia Appendix 1 contains results for both train and test splits and Multimedia Appendix 2 [2,11,17-32] contains de-identification system details. All reported timing results include both train and test splits.

### Deidentification Systems

Our initial set of systems was constrained by an implementation science framing of the problem. As researchers invested in reproducible science, we need to understand the best tools for facilitating ethical data sharing, but our primary research area may not be related to deidentification. We need a resource for evaluating scalable, off-the-shelf systems that do not require additional model training or fine-tuning. Concretely, this framing restricts us to freely available systems that can be run programmatically on a local server without any requirements to annotate a local data set. A total of 6 systems meet these requirements: CliniDeID (version 1.6.1) [17,18], deid by Massachusetts Institute of Technology (version 1.1, now available via PhysioNet) [2,19], MITRE Identity Scrubber Toolkit (MIST; version 2.0.4) [20-22], NeuroNER (commit 3817fea on GitHub) [23,24], National Library of Medicine Scrubber (version 19.0403L Linux ×86 64) [25-27], and Philter (commit 780da99 on GitHub) [11,28]. All 6 of these systems run on English clinical notes, read in files from disc, write annotated files indicating identified PII mentions to disc, can be run from the command line on a Linux server, and have been run using the latest available version. System reporting will be in alphabetical order. Refer to Multimedia Appendix 2 for more details on each system’s configuration and use.

### Metrics

We evaluated systems in terms of both timing and binary classification performance metrics. For timing purposes (reported in the *Timing Results* subsection), each system processed 1 corpus at a time on the same Red Hat Linux server. This evaluation pipeline was the only program beyond routine background processes running on the machine. We used the real (ie, wall clock) time generated by the command line tool time. For performance metrics (ie, encompassing the 3 *Performance Results* subsections in the *Results* section), we used the Evaluation Tool for Unstructured Data and Extractions (ETUDE) to score each system by corpus pairing. ETUDE uses definitions from a configuration file for each of the reference and system outputs to determine how each “native” annotation category is represented in the corpus. These native categories are then mapped in the same configuration file to “scoring value” categories. Drawing from an example depicted in Figure 1, the 2016 native categories of “Phone” and “Fax” are mapped in the 2016 configuration file to the “PhoneFax” scoring value category for tier 1 entries. The configuration files allow us to separate the logic for annotation extraction from annotation alignment matching.

ETUDE also provides several alignment matching algorithms for determining which set of annotations are considered a match between the reference and system outputs. We focused on 3 of these matching algorithms for this study: “exact,” “partial,” and “fully contained.” Exact matching requires that the character offsets of 2 annotations be the same to count as a match. Partial matching only requires that any part of the 2 annotations overlap to be considered a match. Finally, fully contained matching requires the system output annotation to at least cover the entirety of the reference annotation to count as a match. The system output annotation can include more text before or after the extent of the reference annotation but not less. The intuition relevant to deidentification is that if the system PII output annotation fully contains the reference PII annotation, then we know that no PII is leaking, as is potentially possible for a partial match of annotations.

The final evaluation feature we used in ETUDE was the ability to collapse all patterns into a single category for evaluation purposes. Thus, for some of our evaluations, we tracked both annotation and category. For other evaluations, we only tracked the annotations and ignored the differences in annotation category.

In the end, ETUDE generated counts for true positives, false positives, and false negatives. From these counts, we calculated precision, recall, and *F*_1_-score values (ie, the harmonic mean of precision and recall). Given the sensitive nature of deidentification, we give primacy to recall in our reporting in the main body of this paper but include all 3 values in Multimedia Appendix 1. The *F*_2_-score, which gives more weight to recall than precision, can also be generated by ETUDE.

## Results

### Processing Pipeline

**Figure 3:**
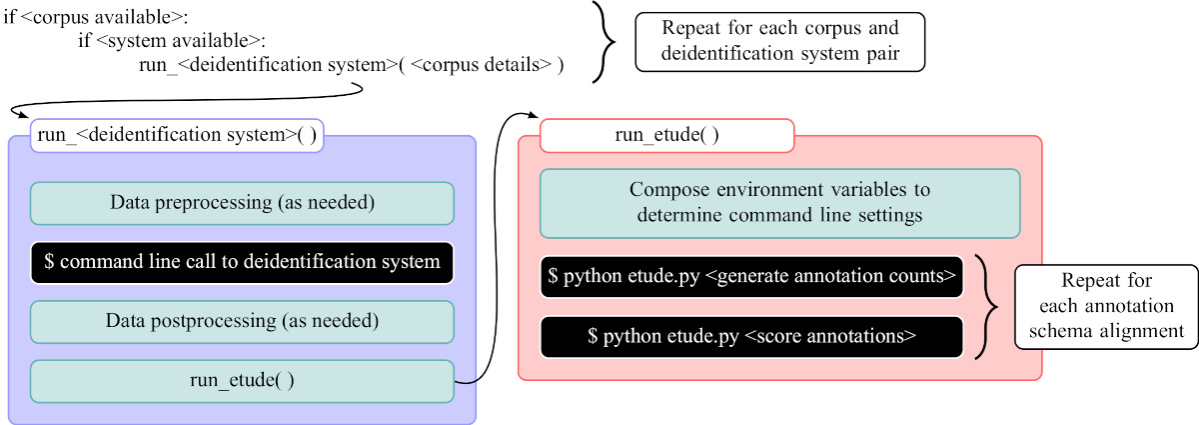
A block diagram showing pseudocode for the logic of running corpus and deidentification system pairs along with the major processes required to be defined for adding new systems.

Expanding on the work of our previous comparison of 3 deidentification systems [33], we developed a larger and more flexible pipeline for using a set of off-the-shelf deidentification systems to process a set of corpora and then scoring all system output at a range of PII category granularities, as shown in the block diagram in Figure 3. The core of this pipeline rests on command line shell scripts with configurable custom functions for processing any given corpus with a given deidentification system. Specifically, the core shell script uses environment variables to set input and output folders for each corpus, running folders and Python environments for each deidentification system, and flags for which set of corpora and systems should run on any given instantiation. The processed output for a system and corpus combination is written to disc in a given folder, allowing for repeated evaluation loops by ETUDE [13,14], a freely available open-source tool developed by the first author. Each evaluation loop uses different configuration settings to highlight different tiers of categories, different alignments of categories between reference and system, and different annotation alignment algorithms. Adding a new deidentification system requires adding a few simple shell commands to preprocess files (as expected by the system), to run the system from the command line with all parameters fully specified, and to postprocess files (if they are not in a format already supported by ETUDE). A new corpus or deidentification system may require creating a new annotation schema mapping file, if the schema is not already covered by those shown in Figure 1. As ETUDE, all shared task corpora, and all deidentification systems are already publicly available, we also released our shell scripts and R-based evaluation scripts on Codeberg and GitHub to allow for near-complete reproducibility of this study [34,35]. System-specific settings, in terms of explicit configuration files or command line flags and settings, are also included in these repositories.

### Timing Results

The 8 timed trials for each system (ie, 4 corpora with 1 train and 1 test split) are summarized in Table 1. We report both the seconds per note (for which a lower value is better) and notes per second (for which a higher value is better). MIST is the fastest with an average of 24.57 (SD 26.23) notes per second and is an order of magnitude faster than all systems except Scrubber, which averages 8.56 (SD 3.54) notes per second. CliniDeID is the slowest with an average of 1 (SD 0.38) note per second. Deid, NeuroNER, and Philter are all marginally faster at an average of 1.41 (SD 0.60), 1.28 (SD 0.48), and 1.36 (SD 0.64) notes per second, respectively.

**Table 1:** Minimum, mean, SD, and maximum processing times for each deidentification system derived from the real time (ie, wall clock) value given by the Linux time utility^a^.

System	Seconds per note			Notes per second		
	Values, minimum (best)	Values, mean (SD)	Values, maximum (worst)	Values, maximum (best)	Values, mean (SD)	Values, minimum (worst)
CliniDeID	0.65	1.19 (0.59)	2.14	1.54	1.00 (0.38)	0.47
deid	0.44	0.85 (0.39)	1.41	2.28	1.41 (0.60)	0.71
MITRE Identity Scrubber Toolkit	0.01	0.08 (0.07)	0.19	80.85	24.57 (26.23)	5.37
NeuroNER	0.52	0.91 (0.41)	0.16	1.93	1.28 (0.48)	0.64
National Library of Medicine Scrubber	0.08	0.14 (0.06)	0.23	13.62	8.56 (3.54)	4.44
Philter	0.45	0.97 (0.62)	1.94	2.24	1.36 (0.64)	0.51

^a^Each data point reflects a single corpus split (eg, the 2006 train split vs the 2006 test split).

### Performance Results Across All Categories Based on Partial Matching

**Figure 4:**
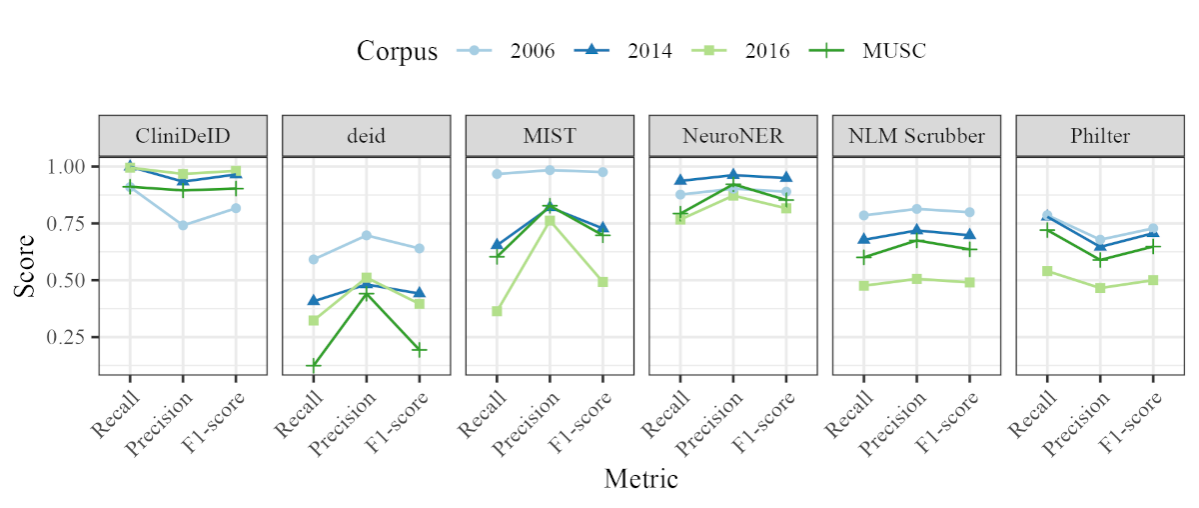
Recall, precision, and F1-score values for partial match annotation alignment wherein all personally identifiable information categories are collapsed into the broad tier 0 of “All Categories.” MIST: MITRE Identity Scrubber Toolkit; MUSC: Medical University of South Carolina; NLM: National Library of Medicine.

For our initial performance metric evaluation, we focused on each system’s overall ability to identify PII, regardless of the category. In the terminology of ETUDE, we collapsed evaluation across all categories. Figure 4 presents the recall, precision, and *F*_1_-scores for each system against each corpus using the partial match alignment (this is the most generous evaluation possible).

The u-shaped curves for CliniDeID and Philter indicate that both these systems prioritize recall over precision. In contrast, the other 4 systems consistently have higher precision scores than recall scores. CliniDeID and NeuroNER show the highest scores across all corpora. Deid and MIST have the largest variance between corpora.

### Performance Results for Specific Tier 1 Categories Based on Partial Matching

**Figure 5:**
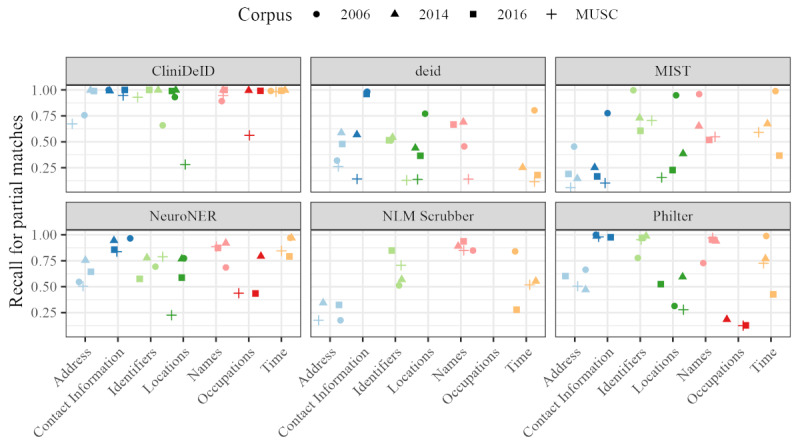
Recall values for partial match annotation alignment broken down into 7 bins for each of the major personally identifiable information groupings in the tier 1 categories. MIST: MITRE Identity Scrubber Toolkit; MUSC: Medical University of South Carolina; NLM: National Library of Medicine.

As different categories of PII are differentially sensitive, we wanted to delve deeper into the specific performance of systems with respect to the 7 tier 1 categories shown in Figure 1. For this analysis, we report the recall for partial annotation matching in Figure 5. Precision and *F*_1_-score values are available in Multimedia Appendix 1. However, the nature of the limited categories generated by deid and Philter means that precision values for these systems are not truly meaningful.

CliniDeID shows more between-corpus variance than between-category variance and overall performs most consistently well. NeuroNER performs the next best across all categories, although “Contact Information,” “Names,” and “Time” are clearly better identified than the other 4 categories. Philter shows the same facility with “Contact Information” and “Names.” Deid shows a large between-corpus variance but is generally consistent across categories. “Occupations” is not included as deid, MIST, and Scrubber do not extract that category. MIST identifies “Address” and “Contact Information” categories at a lower rate than other categories. Similarly, Scrubber identifies “Address” categories at a lower rate and does not extract any “Contact Information” annotations.

### Performance Results for Names Based on Fully Contained Matching

**Figure 6:**
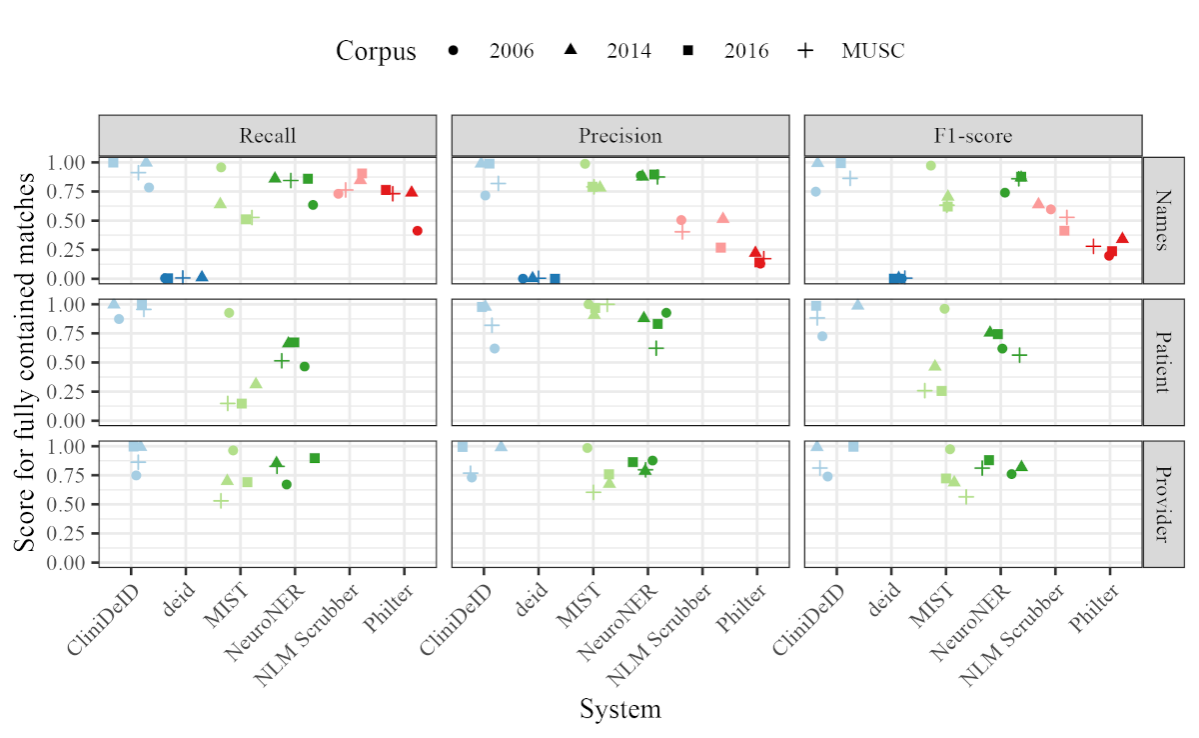
Recall, precision, and F1-score values for fully contained match annotation alignment. The tier 1 “Names” category collapses “Patient,” “Provider,” “Relative,” and “Other Person” names. The tier 2 “Patient” and “Provider” evaluations are given for the 3 systems that distinguish between name categories. MIST: MITRE Identity Scrubber Toolkit; MUSC: Medical University of South Carolina; NLM: National Library of Medicine.

As names are perhaps the most sensitive and unique PII category, we further analyzed the tier 1 “Names” category and the tier 2 categories of “Patient” and “Provider” for those systems that distinguish between these categories. For this analysis, we used the stricter fully contained annotation matching algorithm. Figure 6 summarizes the recall, precision, and *F*_1_-score values across systems and corpora.

No strong corpus-specific trends show up in these results. MIST and NeuroNER have worse recall for identifying patient names than provider names, while CliniDeID does not appear to treat them differently. MIST and NeuroNER also have worse recall than precision for patient names but, similar to CliniDeID, no obvious difference between the 2 metrics for provider names.

The gap between recall and precision is reduced for MIST and NeuroNER at the tier 1 “Names” level, which implies that some (but not all) of the tier 2 performance issues are due to patient names being incorrectly flagged as provider names. CliniDeID is consistently high for both recall and precision at tier 1. The deid system performs the worst for this evaluation, although its scores for “Names” are not so low for the partial matching evaluation in the *Performance Results for Specific Tier 1 Categories* subsection. This performance discrepancy indicates that deid does extract names reliably but does not extract large enough annotations to fully identify the relevant PII. Scrubber and Philter show much higher recall than precision for tier 1 “Names.” As noted previously, Philter’s precision for this evaluation is expected to be very low as the native system category relevant to “Names” includes all PII categories except dates. It is not obvious why Scrubber’s precision is low.

## Discussion

### Overview

As expected, given the complexity of the deidentification domain and feature variability in deidentification tools, we found that that none of the 6 systems uniformly outperformed the others across corpora and PII tag categories. CliniDeID, NeuroNER, and Philter generally outperformed the older (and not actively maintained) systems, but they also each have their performance drawbacks or limitations. Our evaluation framework was helpful in identifying these strong and weak facets, which, in turn, helped us identify additional aspects to include in future releases of the framework, as discussed in the next subsection.

### Principal Findings

The principal findings of this study fall into 3 major classes: the evaluation pipeline implementation, the category mapping for bridging analyses across corpora or deidentification systems, and the evaluation results for the 6 off-the-shelf systems. The evaluation pipeline implementation includes many reusable components, including the shell scripts for passing corpus notes through systems, the ETUDE configuration scripts for scoring system outputs against reference annotations, and R scripts for plotting results of these analyses. The code for replication of the workflow is available via Codeberg and GitHub public code repositories [34,35]. This code base can be used to increase reproducibility between studies on deidentification systems, lower the barrier to fine-tuning or retraining systems, and increase portability of evaluation across data sets and systems. The ETUDE configuration scripts provide a means for encoding and leveraging the PII category cross-mapping displayed in Figure 1. The cross-mapping can also be used to help compare the coverage of different deidentification systems with an eye to identifying the validity of different deidentification systems for different use cases (eg, when some categories of PII are known a priori to be either absent from or very frequent in a data set). Finally, our analysis of the text deidentification systems themselves yielded mixed results. CliniDeID and Philter had the highest recall for select categories, while CliniDeID and NeuroNER had the highest recall across all categories. We also found large differences in performance when comparing partial matches for PII categories to fully contained matches. The tabular data used to generate all tables and figures in this study have been included in Multimedia Appendix 1 to allow for finer-grained evaluation of the results.

### Limitations

As discussed in the Introduction section, we focused our analysis on off-the-shelf models. CliniDeID, MIST, and NeuroNER can be retrained, while National Library of Medicine Scrubber and Philter have dictionaries that can be customized to a local environment. Thus, while our framework provides a means to easily measure the average baseline performance (across multiple corpora), more work is required to ascertain the average potential performance for each system (after retraining). Fortunately, each retrained model would only require a limited pass through the evaluation framework but would not require reprocessing and reevaluating all systems against all corpora. To further facilitate this use case, future iterations of this tool will make it even easier to add new corpora, systems, and evaluation configurations. Specifically, a more robust implementation of the pipeline would leverage reproducible workflow tools such as the targets R package [36]. We have released a related implementation using the targets R package for evaluating algorithmic bias in deidentification systems [37]. Xiao et al [38] provide an alternate approach using 100 synthetic templates imitating realistic contexts for PII as it occurs in unstructured clinical notes. For researchers who do not have the infrastructure or privacy needs of data sets with PII, other reproducible pipelines and shared evaluation frameworks, such as NLP Sandbox, exist [39].

In addition, we have not included all the essential evaluations for deciding between deidentification systems and will continue to expand our result set. For instance, we did not account for system start-up time. A system that takes 1 second per note for 1 or 10 million notes has different usability than a system that takes 0.1 second per note after loading models for 10 minutes. Another evaluation complication is how to equitably compare systems with significantly different input or output features. For instance, CliniDeID resynthesizes annotations by replacing the original PII with similar surrogate values, which likely slows down the overall pipeline compared to a system such as deid that does not track PII category in a manner accessible to a standard user. Similarly, TiDE (Text DEidentification) [40] meets all the aforementioned system requirements but uses prefilled PII details to boost performance. That is, when running a note through TiDE, one can provide the system with the patient’s name, known providers, and other relevant PII that are likely to show up in a note. While this seems like a good plan at the system level, we need to develop a more complex evaluation pipeline to accommodate for the given information and to disaggregate results based on whether TiDE has correctly annotated known PII versus unknown PII (eg, a patient’s nickname or misspelled name and a relative’s name). TiDE was not evaluated and compared in this study for this reason, among others.

Finally, we relied on the mapping in Figure 1 to perform our evaluations, which, in turn, relies on a manual mapping built using experts’ knowledge. Kim et al [41] evaluated a successful automated category mapping algorithm on deidentification concepts, which could be used as an alternate means for cross-mapping categories between corpus annotations and system outputs.

### Comparison With Prior Work

Previous studies have included many of the same individual components that we present as novel in this study but, to the best of our knowledge, none have included all the components together. For instance, a category mapping between the 2014 and 2016 data sets was released at the time of the canonical paper on the 2016 shared task [9]. That same year, the researchers who developed Philter [42] released a mapping between the HIPAA Safe Harbor categories, the i2b2 2014 corpus annotations, and the categories redacted in the public release of the Multiparameter Intelligent Monitoring in Intensive Care II corpus [43,44]. The mapping we present in Figure 1 includes more corpora and extends the mapping to the unique set of categories used by each deidentification system, which greatly extends the utility of this new mapping for the community as a whole. Furthermore, our evaluation tool (ie, ETUDE), along with the published configuration files, provides the means to easily evaluate different category groupings without reannotating the data sets.

Most prior studies do not explicitly mention details of the evaluation (eg, whether they use token counts and exact match character offsets) unless they are using the standard evaluation script released with the 2016 corpus [9]. While the widespread use and public availability of this script are a boon to reproducible science, the script’s limited options, hardcoded categories, and fixed file format are a bane. Most prior studies report the precision, recall, and *F*_1_-score, while a select few report the *F*_2_-score [11,45] or both [46]. We reported the *F*_1_-score, but ETUDE can easily be configured to report the *F* measure for any arbitrary β value. The relative processing speeds (Table 1) for deidentification systems is also rarely reported [33]. A few prior studies have included specific annotation guidelines, including examples and edge cases, to help clarify the underlying ground truth intended by category labels [8,25,26]. Finally, our own work and many of the other researchers and developers cited in this study have relied on the same standard deidentification data sets released via the i2b2 or CEGS N-GRID shared tasks. For less common but still publicly available data sets, the developers of NeuroNER evaluated their system against the CoNLL 2003 shared task on named entity recognition [23], and Xiao et al [38] recently released a new smaller set of notes based on MIMIC-IV. We are not aware of any other study with a publicly released evaluation framework that includes all steps from initial corpus processing through plotting evaluation results.

### Conclusions

We present a reusable and extensible evaluation framework applied to deidentification systems for clinical unstructured notes. We release the tool for other researchers to reduce the overhead in testing new systems and new corpora, a critical and common task within the clinical NLP community, especially for researchers who wish to ethically share data without leaking PHI.

From this initial case study, we found an order of magnitude difference in terms of processing speed between the fastest and slowest systems and that no single system out of the 6 uniformly outperformed the others across corpora and PII categories. Instead, a richer tapestry of recall and precision trade-offs emerged for different PII categories and groups of PII categories. We hold that a single evaluation pipeline across multiple systems and corpora allows for more nuanced comparisons between systems and serves as a boon to the clinical NLP community.

## Appendix

Multimedia Appendix 1 CSV data files used to generate figures.

Multimedia Appendix 2 Details on each of the 6 deidentification system’s release information (eg, version number), configuration, and use.

## Abbreviations

CEGS N-GRID: Centers of Excellence in Genomic Science Neuropsychiatric Genome-Scale and RDOC Individualized Domains
ETUDE: Evaluation Tool for Unstructured Data and Extractions
HIPAA: Health Insurance Portability and Accountability Act
i2b2: Informatics for Integrating Biology and the Bedside
IRB: institutional review board
MIST: MITRE Identity Scrubber Toolkit
MUSC: Medical University of South Carolina
NLP: natural language processing
PHI: protected health information
PII: personally identifiable information
TiDE: Text DEidentification
UTHealth: University of Texas Health Science Center at Houston

## Data Availability

The 2006 and 2014 corpora analyzed during this study are available in the National NLP Clinical Challenges website repository [47]. The 2016 corpus analyzed during this study is not publicly available due to privacy concerns. The Medical University of South Carolina corpus is not publicly available due to privacy concerns, as the data set has not been fully redacted or resynthesized to remove personally identifiable information. Code for replication of the workflow is available via Codeberg and GitHub public code repositories [34,35].
