# Supplementary material for: An Extensible Evaluation Framework Applied to Clinical Text Deidentification Natural Language Processing Tools: Multisystem and Multicorpus Study
Source: J Med Internet Res. 2024 May 28;26:e55676. doi: 10.2196/55676 (PMC11167315; doi:10.2196/55676)
Supplement: Multimedia Appendix 2 [file jmir_v26i1e55676_app2.docx]

### Deidentification System Details

CliniDeID (version 1.6.1) was originally a commercial tool developed by Clinacuity, Inc. and is now freely available on GitHub [17,18]. It uses an ensemble system to combine dictionary look-up, rule-based, shallow learning, and deep learning algorithms. It, along with MIST, natively supports resynthesis (also called "hiding in plain sight")[29] of PII mentions after their identification. We evaluated 19 distinct output categories, as shown in Figure 1.

MIT’s deid (version 1.1, now available via PhysioNet) system is the oldest that we evaluated [2,19]. Detected PII is returned as a list of offset mentions without any distinctive categories, as shown in Figure 1. We determined the category mapping based on the prior literature and targeted testing on the train split output. This targeted testing involved scoring the recall performance of deid’s output against each subset of the Tier 1 and Tier 2 categories. We judged a category to not be covered if recall was less than 25%.

MIST (MITRE Identity Scrubber Toolkit, v 2.0.4) uses conditional random fields (CRFs) as its primary algorithm for deidentification [20-22]. We used the “AMIA Deidentification” task, as described in the manual with no additional fine-tuning or training. Of the 11 categories described in the literature, only 8 of them were flagged in any of the corpora. The three “Electronic Address”

categories that were not flagged are marked with an asterisk in Figure 1. While MIST supports resynthesis as an output option, we did not use this customization in our pipeline as resynthesis does not impact our evaluation metrics despite being a critical feature for more secure deidentification.

NeuroNER (commit 3817fea on GitHub) includes a trained bidirectional long short-term memory (BiLSTM) model called ‘i2b2 2014 glove spacy bioes’ [23,24]. This model uses GloVe [30] for its pre-trained word embedding and was fine-tuned for deidentification. We use the model without any additional fine-tuning, although the architecture allows for it. The system outputs 18 labels parallel to the 2014 corpus annotation categories.

Scrubber (version 19.0403L Linux x86 64) is available via the National Library of Medicine (NLM) and uses rules and dictionaries to identify 7 PII categories [25-27]. Two of these categories (“Organization” and “Telecom”) were never tagged in our corpora. Advanced parameters (like the list of “preserved terms” to never de-identify) were not used. Unlike other systems which produce character offset mentions for all flagged PII, Scrubber redacts the PII. The opening line of Moby Dick is rendered “Call me [PersonalName]”. We wrote a script to convert this redacted format into brat standoff [31] format by attempting to recover the mentions of the reacted text (i.e., the PII). In the above example, “Ishmael” covers the characters 9–15. Consecutive redactions (e.g., the “[PersonalName]\n[Address]” format used on envelopes) are merged into a single annotation and a copy of the combined annotations is tagged with both categories. Scrubber is sensitive to the artificial dates used in the 2006, 2014, and 2016 corpora. Specifically, some of the resynthesized dates are too far in the future to reasonably occur in a real clinical note and, thus, Scrubber does not flag them. To avoid this problem, we generated a normalized date corpus equivalent of all three original corpora with future dates replaced with years between 1950 and 2021. Both scripts are available via GitHub in the nlm-scrubber folder (for converting Scrubber's output format into brat) and the i2b2 folder (for generating a normalized date corpus) [32].

Philter (commit 780da99 on GitHub) uses a combination of pattern matching, statistical modeling, block lists, and allow lists to tag PII mentions with 1 of 2 categories [11,28]. As with deid, we used published research descriptions and targeted testing to determine the mapping of category labels.

Each of the 6 systems can be improved in some manner beyond the basic off-the-shelf performance. CliniDeID, Physionet deid, and NLM Scrubber have dictionaries that can be customized. CliniDeID, Physionet deid, and Philter have rules that can be customized. CliniDeID, MIST, and NeuroNER can be re-trained with annotated data.

### Abbreviations

i2b2: Informatics for Integrating Biology and the Bedside

MIST: MITRE Identity Scrubber Toolkit

NLM: National Library of Medicine

PII: personally identifiable information
